# Experimental Implantation of the Generative Glands—Successful Testis Implantation of a Subject Dead Twenty-four Hours—Implantation of an Ovary from a Subject Dead Twenty-three Hours

**Published:** 1914-04

**Authors:** G. Frank Lydston

**Affiliations:** Chicago


### Abstracts and Selections.

### Experimental Implantation of the Generative Glands—Successful Testis Implantation of a Subject Dead Twenty-four Hours—Implantation of an Ovary From a Subject Dead Twenty-three Hours.

BY G. FRANK LYDSTON, M. D., CHICAGO.

Having in mind what has long seemed to me to be the true etiology of dementia precox, the nervous conditions resulting from extensive mutilating operations on the female pelvis, and the possibility of retarding senile changes, prolonging life and increasing efficiency by the administration of the internal secretions of sex-glands in both the male and the female, I have recently made two experiments which seem to me both interesting and of practical value. I have prepared a paper upon the general subject of the therapeutic possibilities of sex-gland implantation, which I will later present to the profession. My reason for presenting experiments which I have made at this time is simply that I wish to immediately establish priority.

Experiment 1. On January 16, 1914, I secured a testis of a suicide, eighteen years of age, dead seventeen hours. Twenty-four hours after the death of the subject I implanted it upon my own right spermatic cord within the scrotal sac, my associate, Dr. Carl Michel, assisting. The technique of the implantation will appear later. On the eighth day thereafter I removed one-half of the implanted gland and found that it had become adherent, as was shown by distinct and firm vascular adhesions. The remainder of the gland had been decorticated prior to implantation and was permitted to remain. This portion of the testis forms at the present time a typically formed movable glandular mass, about the size of a small almond, outlined by palpation. After removal of part of the gland a fistula existed for five weeks, and from this fistula was discharged a muco-sanious secretion, in which immature spermatozoids were found. A complete description of the local and general results of the implantation will be contained in my paper, subsequently to be presented. The progress since the implantation and the results have been inspected by Drs. R. D. MacArthur, W. E. Quine, J. B. Murphy, Carl Michel, Charles E. Caldwell, M. M. Portis, Lee A. Stone, A. C. Crofton, Wallace Blanchard, Henry F. Lewis, J. Cooke-Adams, of Chicago, and Terry M. Townsend, of New York.

Experiment 2. I secured the ovaries of a subject, seventeen years of age, dead by violence for twelve hours. A woman, fifty-nine years of age, suffering from the effects of a total ablation of the uterine appendages sixteen years ago, to whom I explained the theory, methods and dangers of the operation, courageously consented to have me perform the implantation experiment upon her. One of the ovaries of the dead subject was implanted eleven hours later (March 2) in the patient's left labium majus by a technique to be hereafter described. Dr. Carl Michel assisted at the operation. It is too early yet to predict results, but the wound is healing kindly and I have every reason to hope for success from the implantation. The results will later be presented to the profession.

The conclusions which will be submitted in the aforesaid paper will embrace: (1) Selection of appropriate dead subjects as donors of sex-glands; (2) the probability of successful implantation of glands which have been frozen—with due credit to Carrel for his remarkable observations and experiments; (3) the possibility or probability of benefit from implantation of sex-glands in dementia precox, its congeners, impotency and sterility, psychopathia sexualis, chronic diseases of the skin, the disturbances incidental to the menopause, arteriosclerosis, the results of sex mutilations, and retarding senility; (4) the question of permanency of result. In this paper also will appear a plea for such legislative action and education of public sentiment as shall enable us to obtain for experimental and therapeutic purposes the material necessary for sex-gland implantation.—*Bulletin Chicago Medical Society.*

